# Supplementary material for: Oligomeric-solvent engineering of hierarchical hydrogen-bonding networks for multifunctional glass interlayers
Source: Nat Commun. 2026 Mar 7;17:3607. doi: 10.1038/s41467-026-70223-7 (PMC13096342; doi:10.1038/s41467-026-70223-7)
Supplement: Supplementary file 1 — Supplementary Information [file 41467_2026_70223_MOESM1_ESM.pdf]

Supplementary Information

Oligomeric-Solvent Engineering of Hierarchical Hydrogen-bonding Networks for Multifunctional Glass Interlayers

Min Li[1], Longyu Hu[1], Menghan Pi[1], Xiayue Yang[1], Xiaoyu He[1], Wei Cui [1]*, and Rong Ran[1]*

[1]College of Polymer Science and Engineering, State Key Laboratory of Polymer Materials Engineering, Sichuan University, Chengdu 610065, China.

*Corresponding authors:

(W.C.) cuiwei@scu.edu.cn;

(R.R.) ranrong@scu.edu.cn.

This file includes:

Supplementary Figure 1 to Supplementary Figure 39

Supplementary Table 1 and Supplementary Table 2

Supplementary Refence

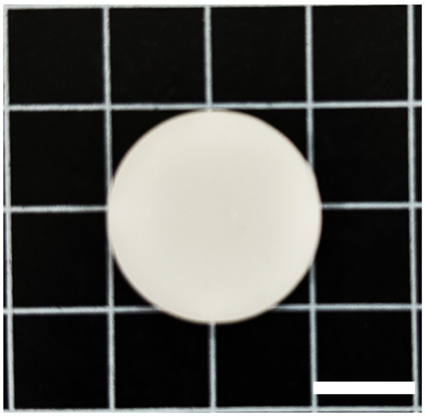

Supplementary Fig. 1. Photographs showing that substituting PEG with H_2O at the same molar ratio (PMAA hydrogel: 0.02 mol MAA and 0.01 mol H_2O) produces a white, hard, and brittle plastic sheet rather than a hydrogel. This result indicates that matching the molar ratio alone is insufficient to reproduce the role of PEG in the PE_xM_y system. H_2O disrupts PMAA–PMAA hydrogen bonding but fails to establish a stable bridging network, whereas PEG oligomers can simultaneously interact with multiple PMAA chains, enabling effective chain separation and network connectivity. Scale bar: 1 cm.

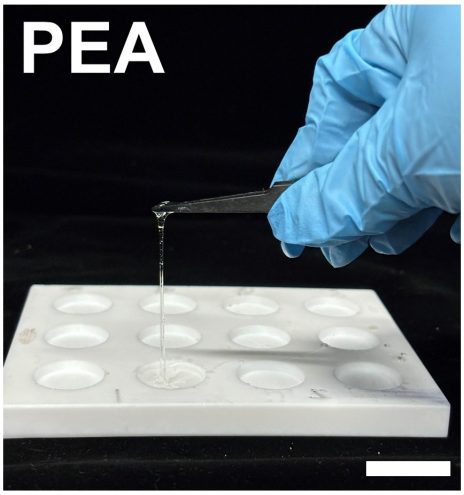

Supplementary Fig. 2. Photographs illustrating the failure of gelation when MAA is replaced by AAc in the PEG-containing precursor solution. This is because the alpha-methyl substituent increases hydrophobicity and stabilizes strong carboxyl to carboxyl associations[1,2]. However, PAAc lacks the alpha-methyl group and therefore prevents gelation in the PEA system, consistent with the loss of the strong-bond tier in the absence of robust self-association among carboxyl groups. Scale bar: 2 cm.

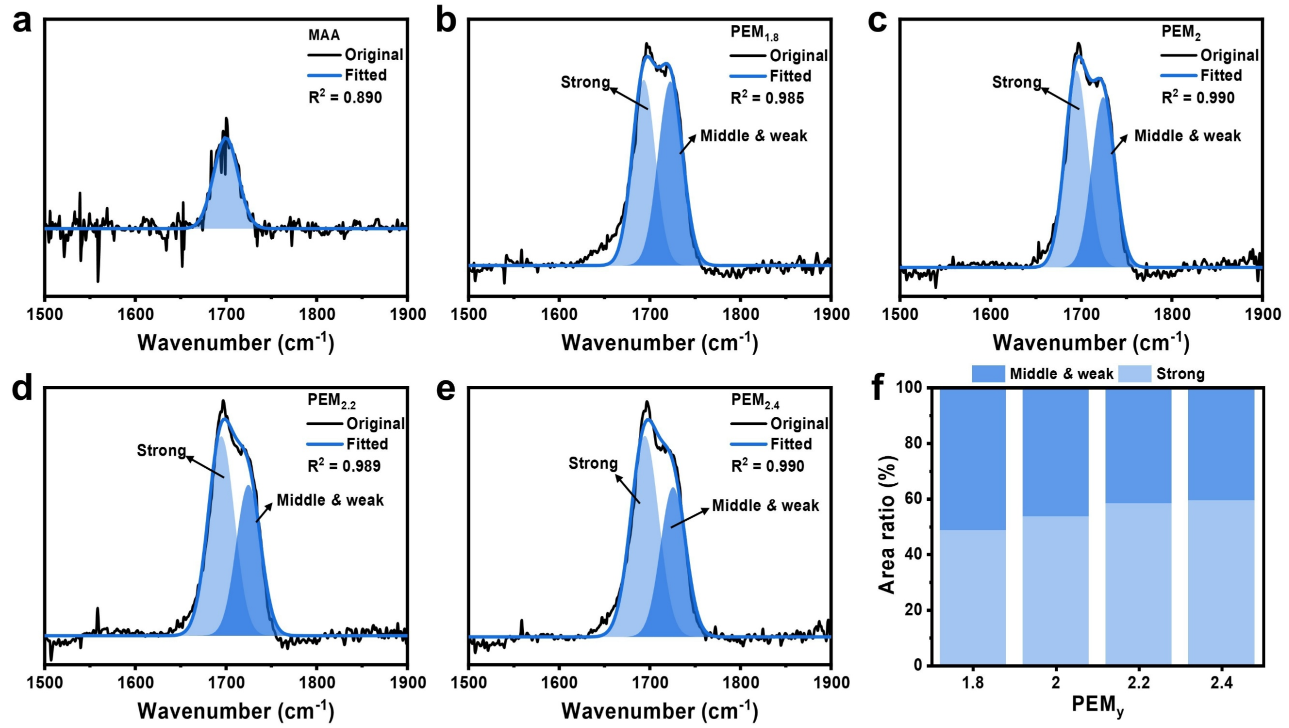

Supplementary Fig. 3. Peak fitting analysis of the carbonyl stretching vibration [ν(C=O)] at 1700 cm^{-1}. a Pure MAA. **b-e** PEM_y gels, y = 1.8, 2, 2.2, and 2.4. **f** Corresponding area fractions of the fitted carbonyl peak.

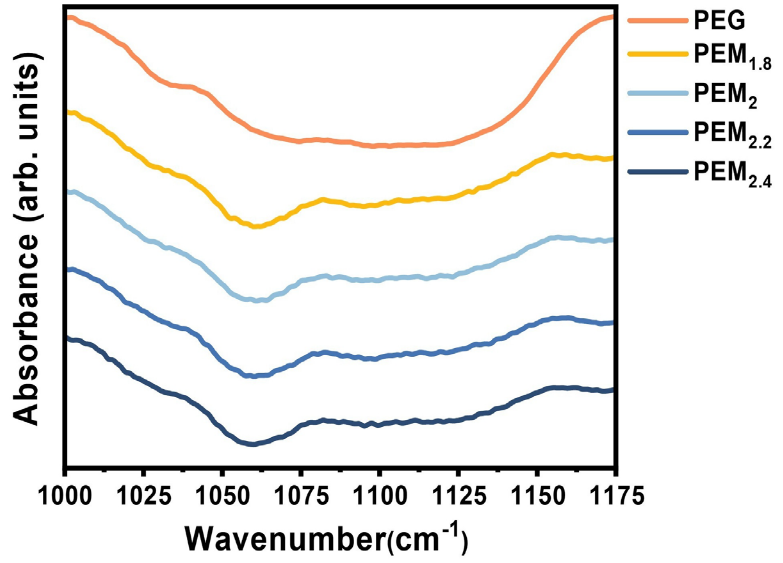

Supplementary Fig. 4. Enlarged FT-IR spectra in the C–O–C stretching region (1000–1100 cm^{-1}) of neat PEG and PEM_y gels.

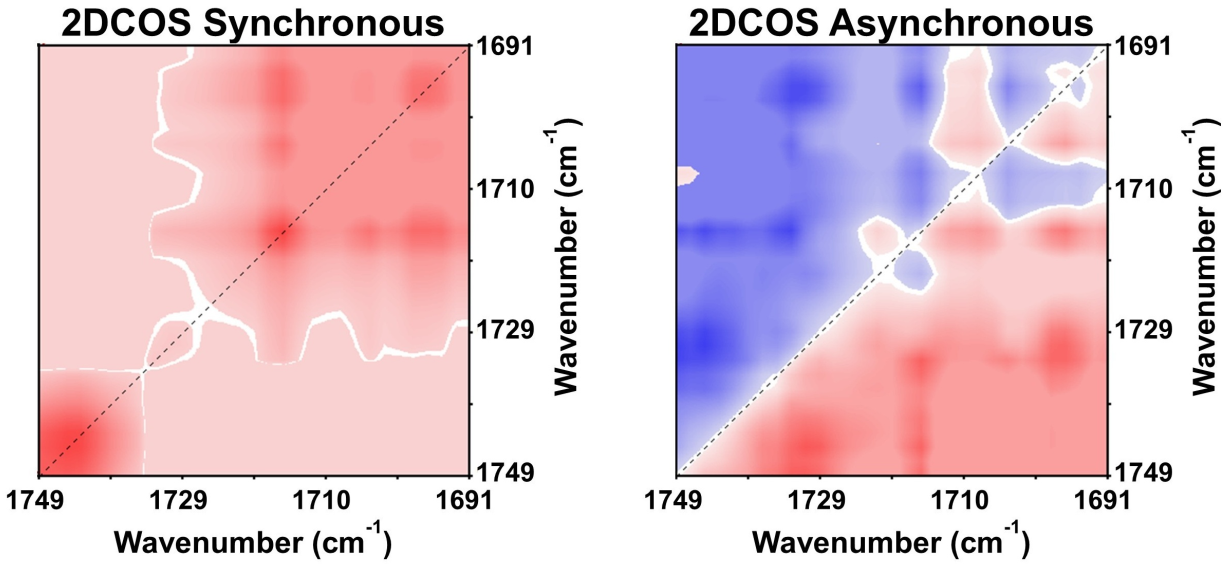

Supplementary Fig. 5. Synchronous and asynchronous 2DCOS spectra of the PEM₂ gel. Red regions indicate positive intensities, while blue regions represent negative ones.

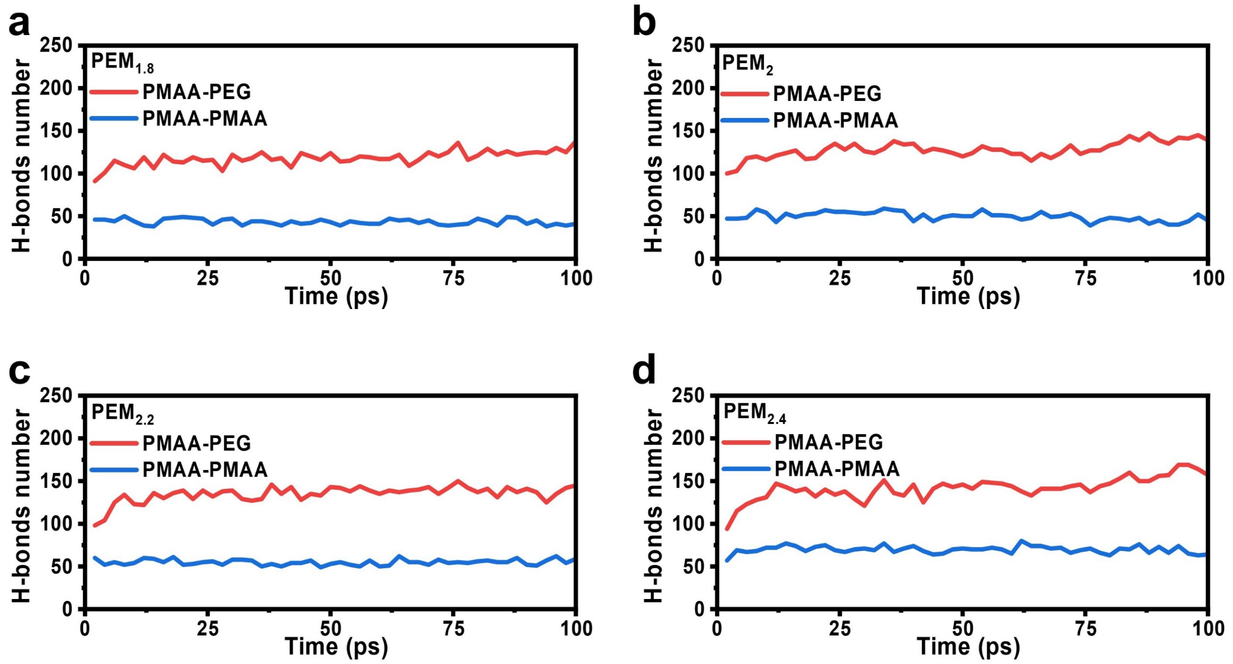

Supplementary Fig. 6. Hydrogen-bond counts in PEM_y gels obtained from molecular dynamics (MD) simulations. a $PEM_{1.8}$. **b** PEM_2. **c** $PEM_{2.2}$. **d** $PEM_{2.4}$.

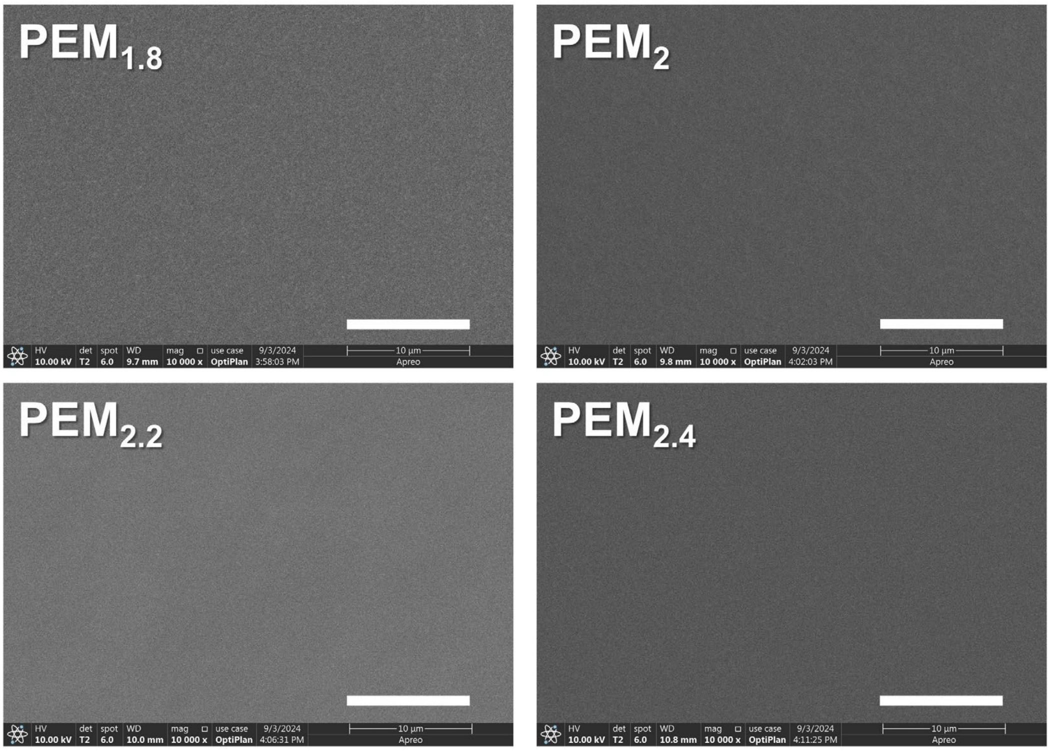

Supplementary Fig. 7. SEM images of all PEM$_y$ gels. Scale bar: 10 μm.

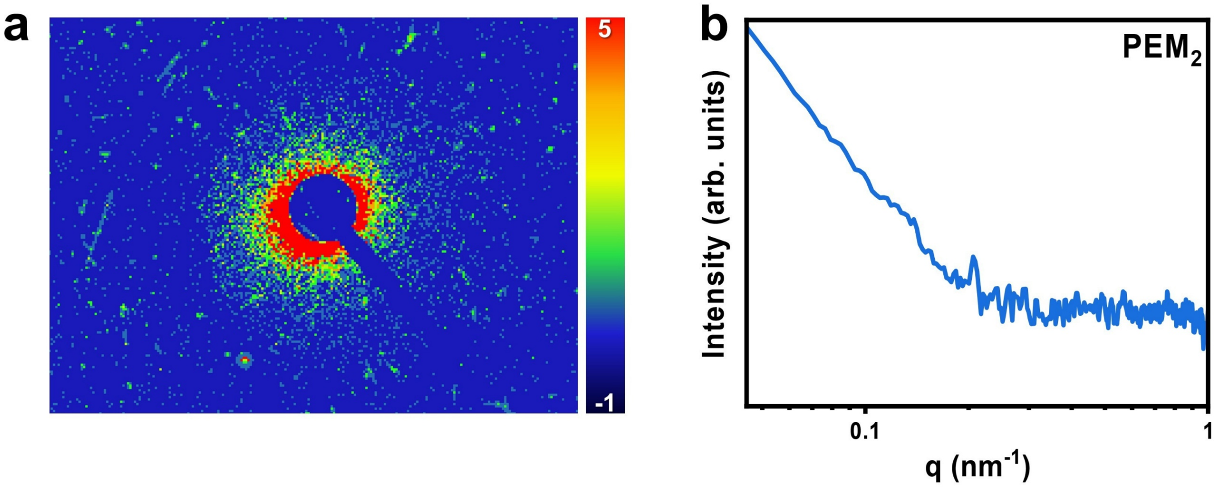

Supplementary Fig. 8. SAXS profiles of the PEM$_2$ gel. a 2D SAXS pattern. **b** 1D scattering intensity profile. The morphological uniformity is further supported by SAXS measurements of the PEM2 gel, which show no scattering rings or correlation peaks in the 1D scattering intensity profile, confirming the absence of phase separation[3].

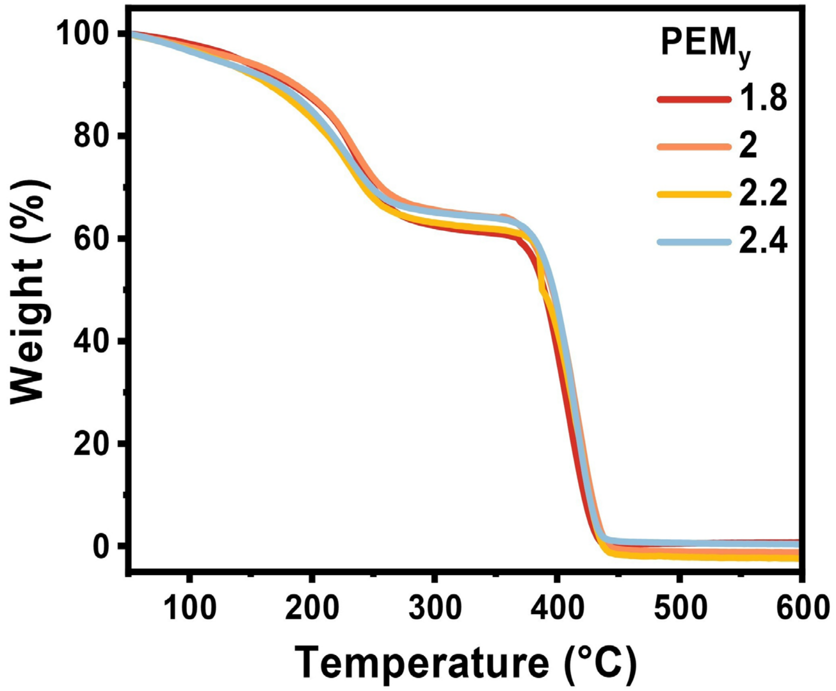

Supplementary Fig. 9. TGA curves of all PEM_y gels.

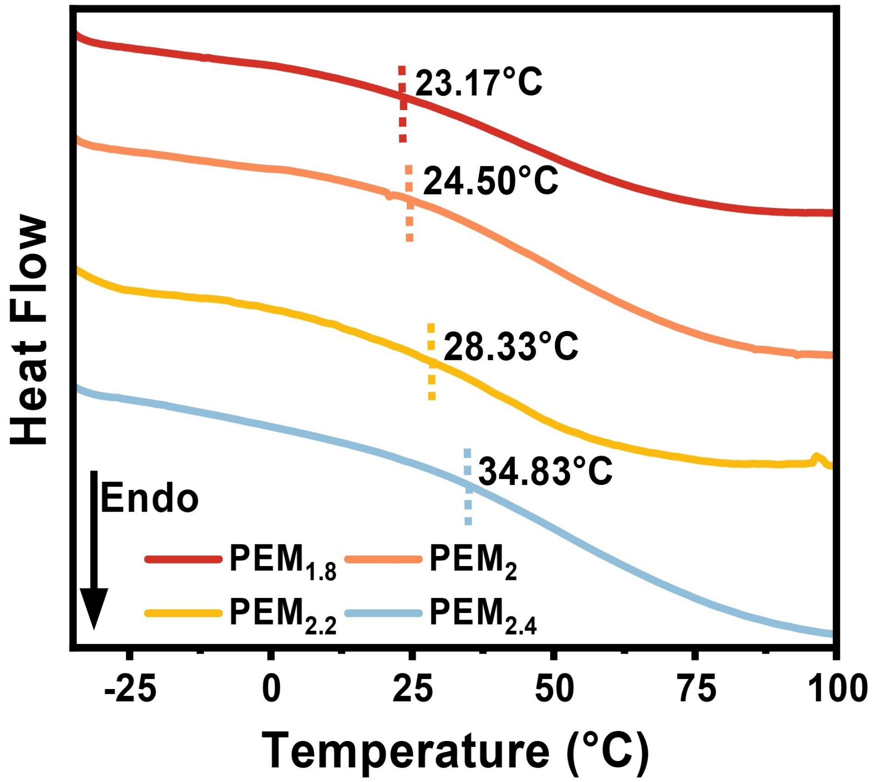

Supplementary Fig. 10. DSC curves of all PEM_y gels.

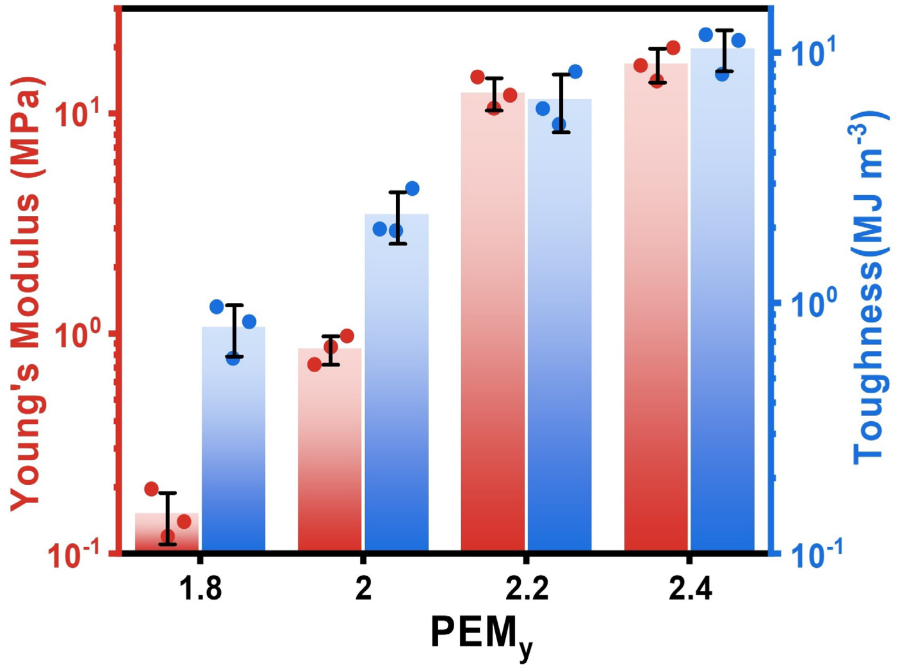

Supplementary Fig. 11. Summary of Young's modulus and toughness values of PEM$_y$ gels. Data are presented as mean values ± SD, n = 3 independent samples. Source data are provided as a Source Data file.

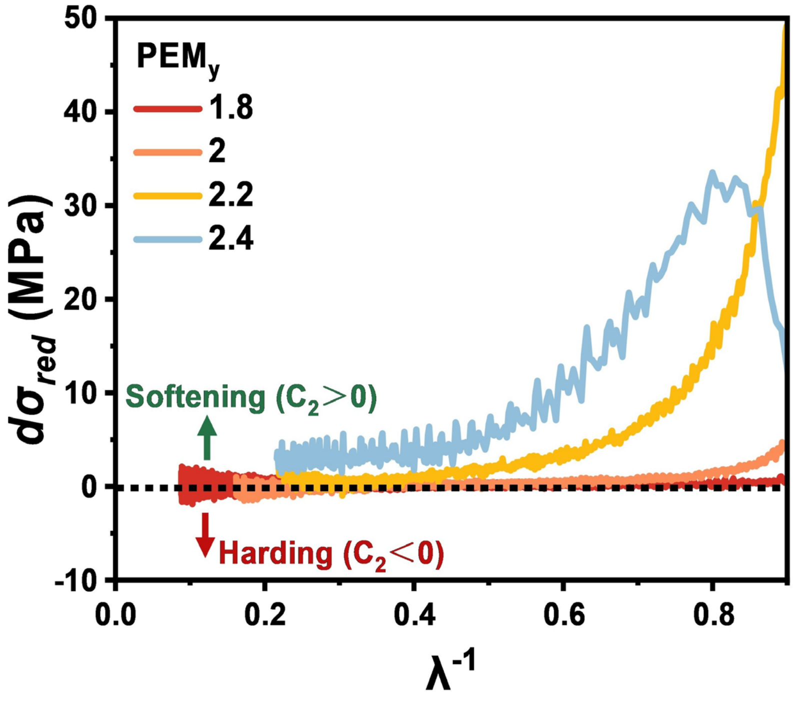

Supplementary Fig. 12. First-derivative Mooney-Rivlin plots of PEM$_y$ gels.

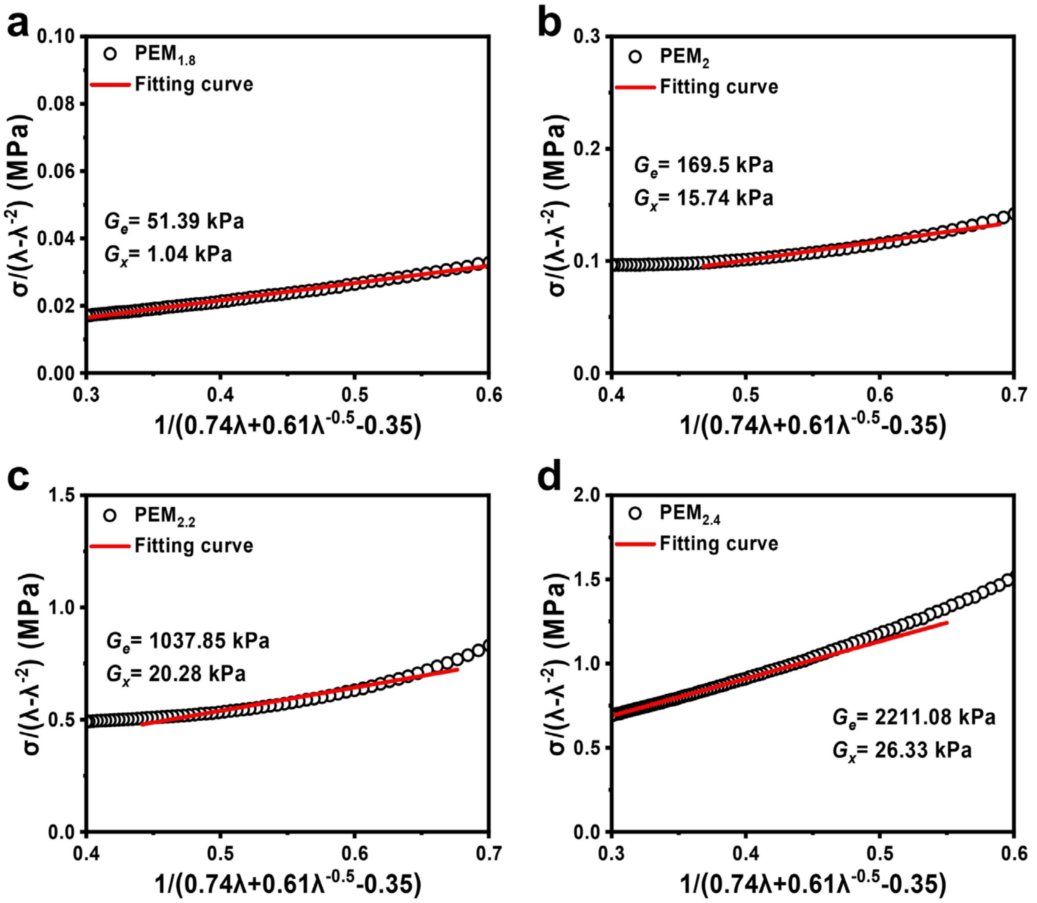

Supplementary Fig. 13. Variations of $\sigma/(\lambda-\lambda^{-2})$ as a function of $1/(0.74\lambda+0.61\lambda^{-0.5}-0.35)$, together with the corresponding linear fit.

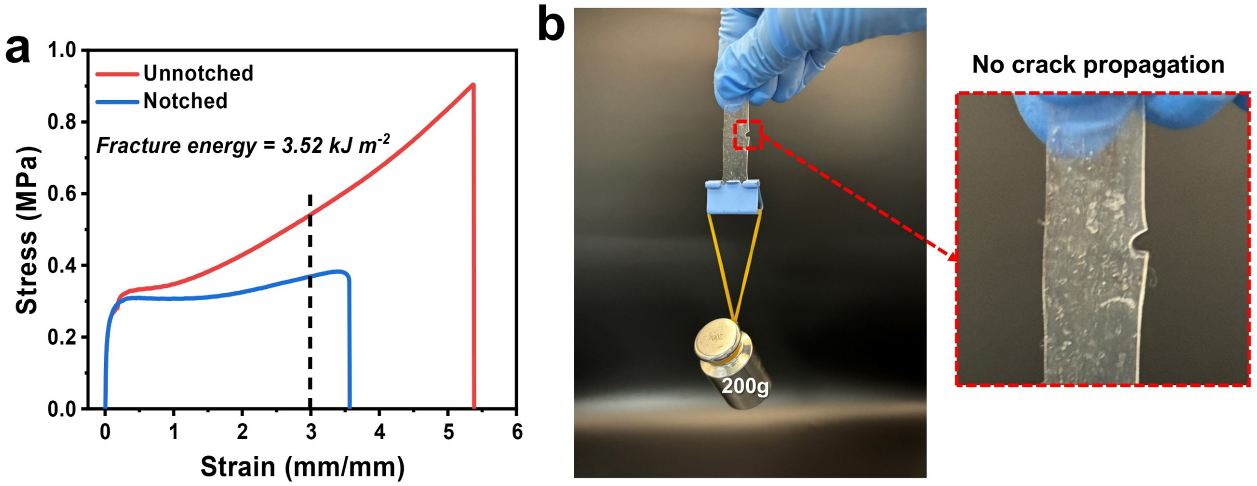

Supplementary Fig. 14. Single-edge notch test for evaluating the fracture energy of the PEM_2 gel. a Tensile stress-strain curves of unnotched and notched PEM_2 gel samples, with the dashed line indicating the strain at crack initiation. **b** Photographs showing the notched PEM_2 gel lifting a 200 g weight without crack propagation.

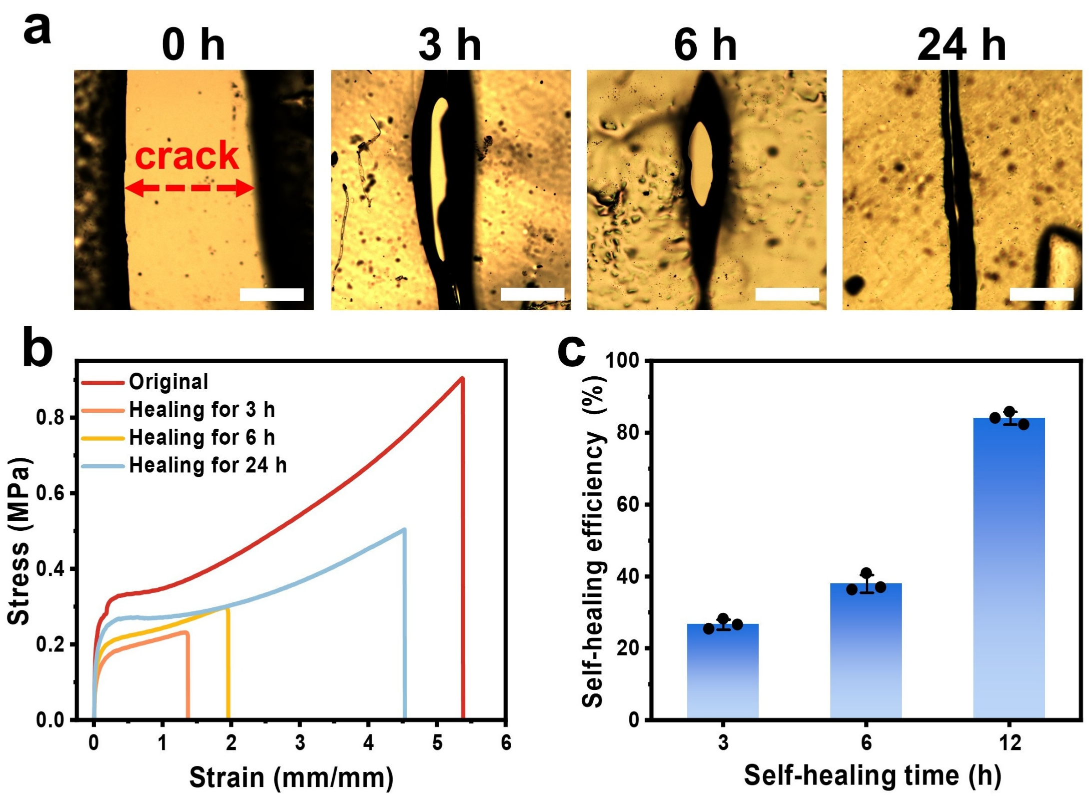

Supplementary Fig. 15. Self-healing of the PEM$_2$ gel. a Optical images of PEM$_2$ gels at different self-healing times. Scale bar: 500 μm. **b** Tensile stress-strain curves of PEM$_2$ gels with different self-healing times. **c** Self-healing efficiency of PEM$_2$ gels at different self-healing times, based on fracture strain recovery. Data are presented as mean values ± SD, n = 3 independent samples. Source data are provided as a Source Data file.

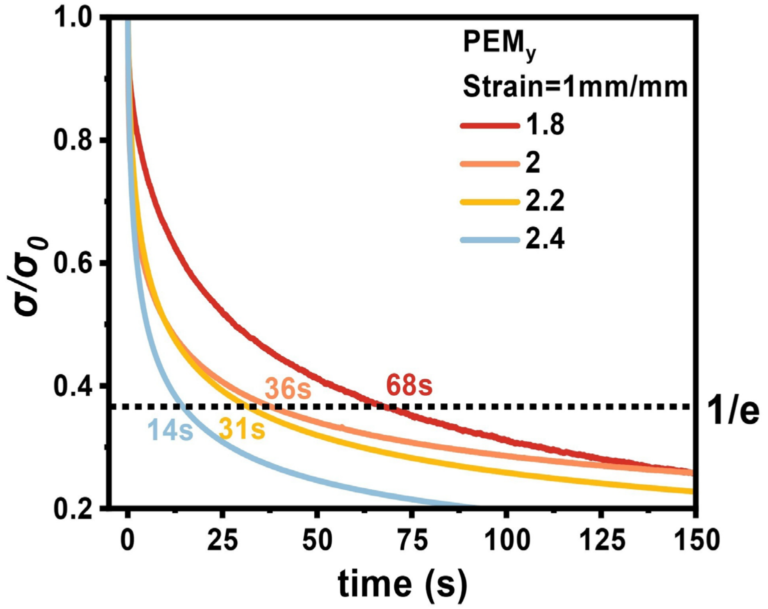

Supplementary Fig. 16. Stress relaxation curves of PEM$_y$ gels measured at a fixed strain of 1 mm/mm.

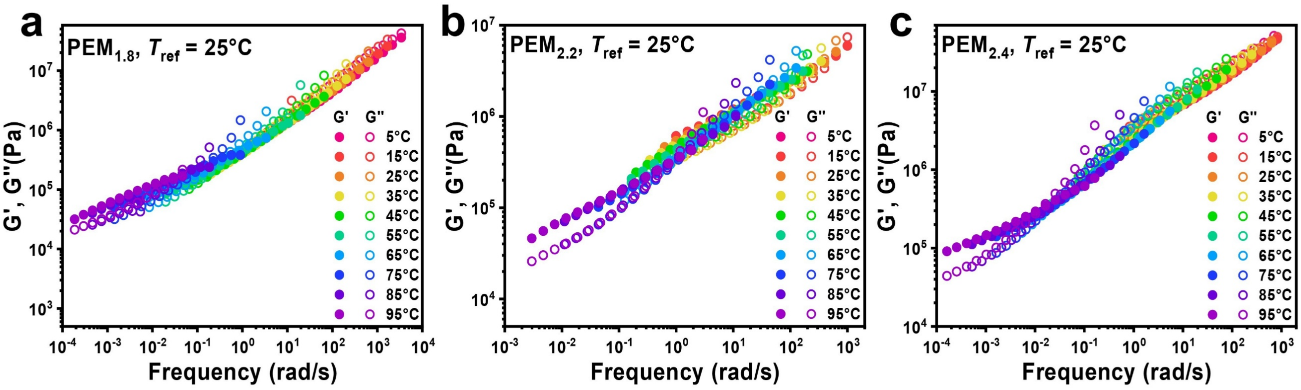

Supplementary Fig. 17. Rheological master curves for PEM$_y$ gels constructed using the time-temperature superposition principle at a reference temperature of 25°C. a PEM$_{1.8}$. **b** PEM$_{2.2}$. **c** PEM$_{2.4}$.

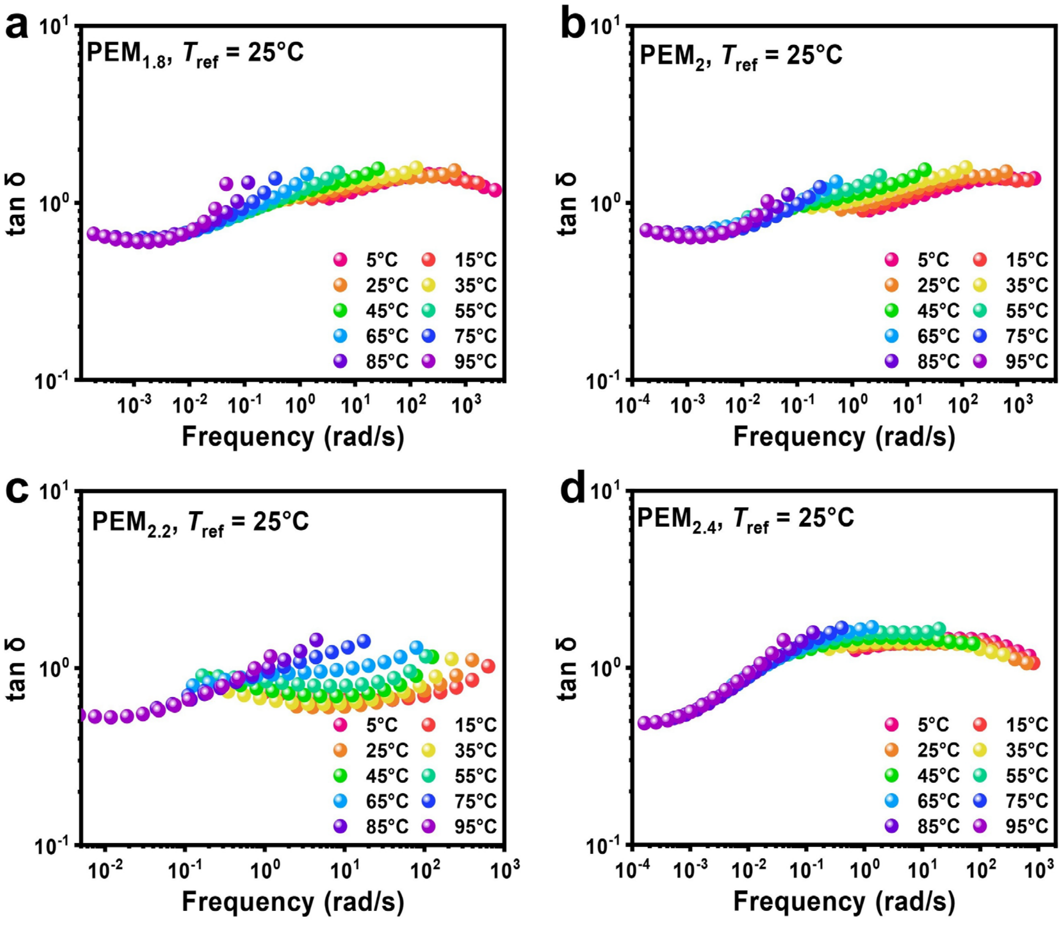

Supplementary Fig. 18. Loss factor of PEM$_y$ gels obtained using the time-temperature superposition principle at a reference temperature of 25 °C. a PEM$_{1.8}$. **b** PEM$_2$. **c** PEM$_{2.2}$. **d** PEM$_{2.4}$.

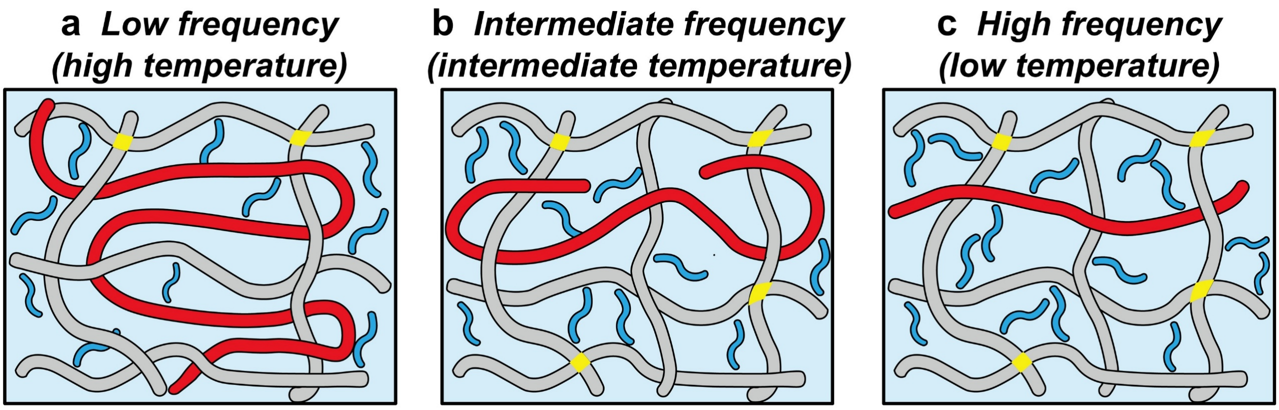

Supplementary Fig. 19. Internal network state of PE_xM_y gels under different shear frequencies.

Supplementary Fig. 19 schematically illustrates the physical origin of the quasi-frequency- and temperature-independent viscoelastic response of PEM_y gels within the framework of time–temperature equivalence and relaxation-spectrum theory.

In viscoelastic polymer networks, the macroscopic balance between elasticity and viscosity is governed by the superposition of multiple relaxation modes with distinct characteristic times. When these relaxation modes are narrowly distributed, energy dissipation is localized near the glass transition, leading to a sharp peak in tan δ. In contrast, a broad and overlapping relaxation spectrum can give rise to a nearly constant loss factor over extended frequency and temperature ranges.

In PEM_y gels, such a broad relaxation spectrum originates from the hierarchical hydrogen-bonding network combined with chain entanglements. At low frequencies (or equivalently high temperatures, Supplementary Fig. 19a), weak and medium-strength hydrogen bonds dissociate first, increasing chain mobility and releasing long network strands. Although the effective density of elastic constraints decreases due to bond dissociation, the liberated chains simultaneously form additional topological entanglements. This compensatory mechanism preserves the balance between energy storage and dissipation, preventing a transition to purely viscous flow. At intermediate frequencies (Supplementary Fig. 19b), partial rupture of weak hydrogen bonds occurs while strong PMAA–PMAA hydrogen bonds and chemical crosslinks remain intact. In this regime, hydrogen-bond dissociation and entanglement formation coexist dynamically, establishing a steady-state viscoelastic response in which the relaxation times of different modes overlap. As a result, both G′ and G″ remain comparable in magnitude. At high frequencies (or low temperatures, Supplementary Fig. 19c), molecular motion is increasingly constrained, suppressing chain reptation and entanglement relaxation. However, the dominance of strong hydrogen bonds and physical crosslinking effectively “freezes” the network on the experimental timescale, sustaining elastic energy storage. Consequently, although individual relaxation processes

are restricted, the collective contribution of long-lived elastic modes compensates for the reduced viscous dissipation.

Overall, the hierarchical hydrogen-bond architecture generates a continuous distribution of relaxation times spanning bond dissociation, chain sliding, and entanglement relaxation. The superposition of these modes underlies the observed G′ ≈ G″ and tan δ ≈ 1 behavior across an ultra-wide frequency and temperature window, analogous in principle to multi-relaxation damping systems, yet achieved here through dynamic supramolecular interactions rather than confined polymer fluids.

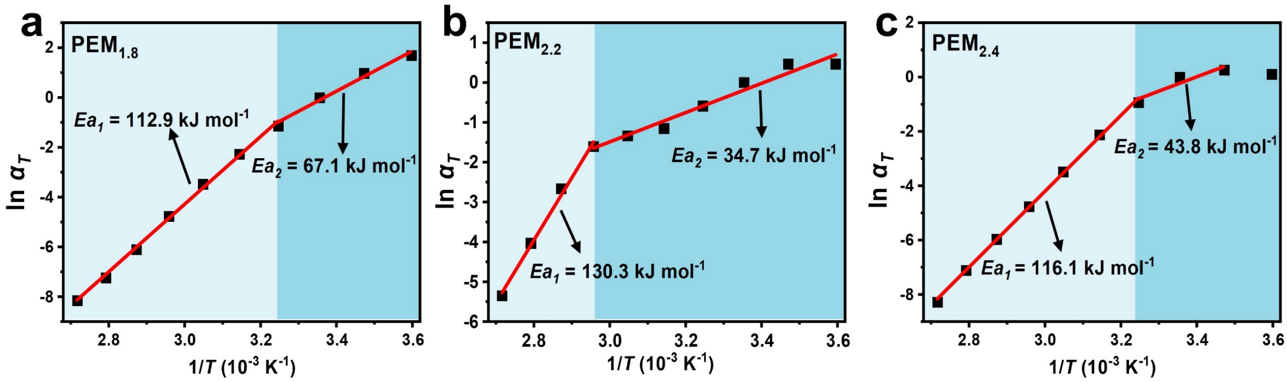

Supplementary Fig. 20. Fitting curves for determining the apparent activation energy of PEM$_y$ gels. a PEM$_{1.8}$. **b** PEM$_{2.2}$. **c** PEM$_{2.4}$.

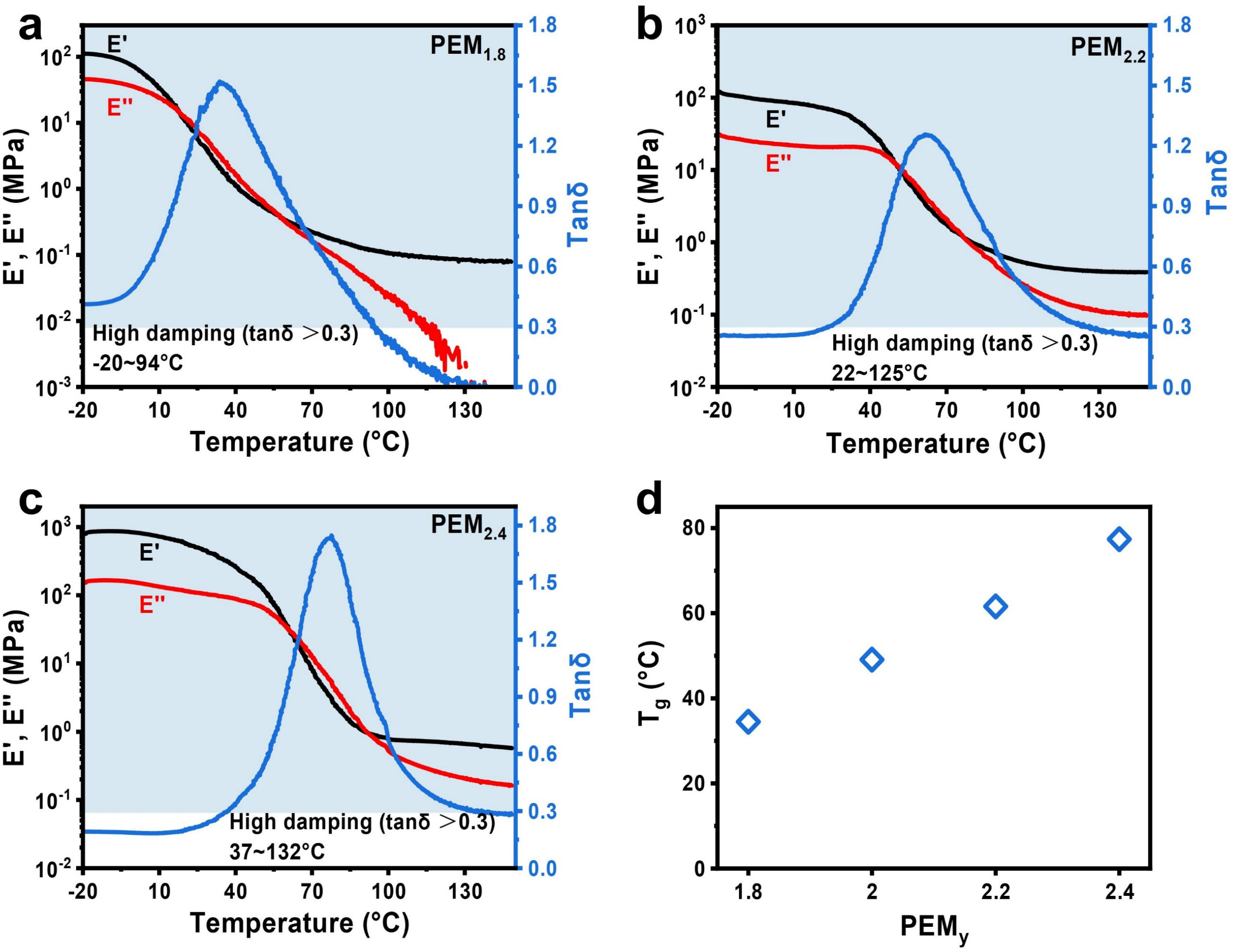

Supplementary Fig. 21. Temperature-dependent DMA results of PEM$_y$ gels between -20 and 150 °C. a PEM$_{1.8}$. **b** PEM$_{2.2}$. **c** PEM$_{2.4}$. **d** Summary of the glass transition temperature (T_g) of all PEM$_y$ gels obtained through DMA test.

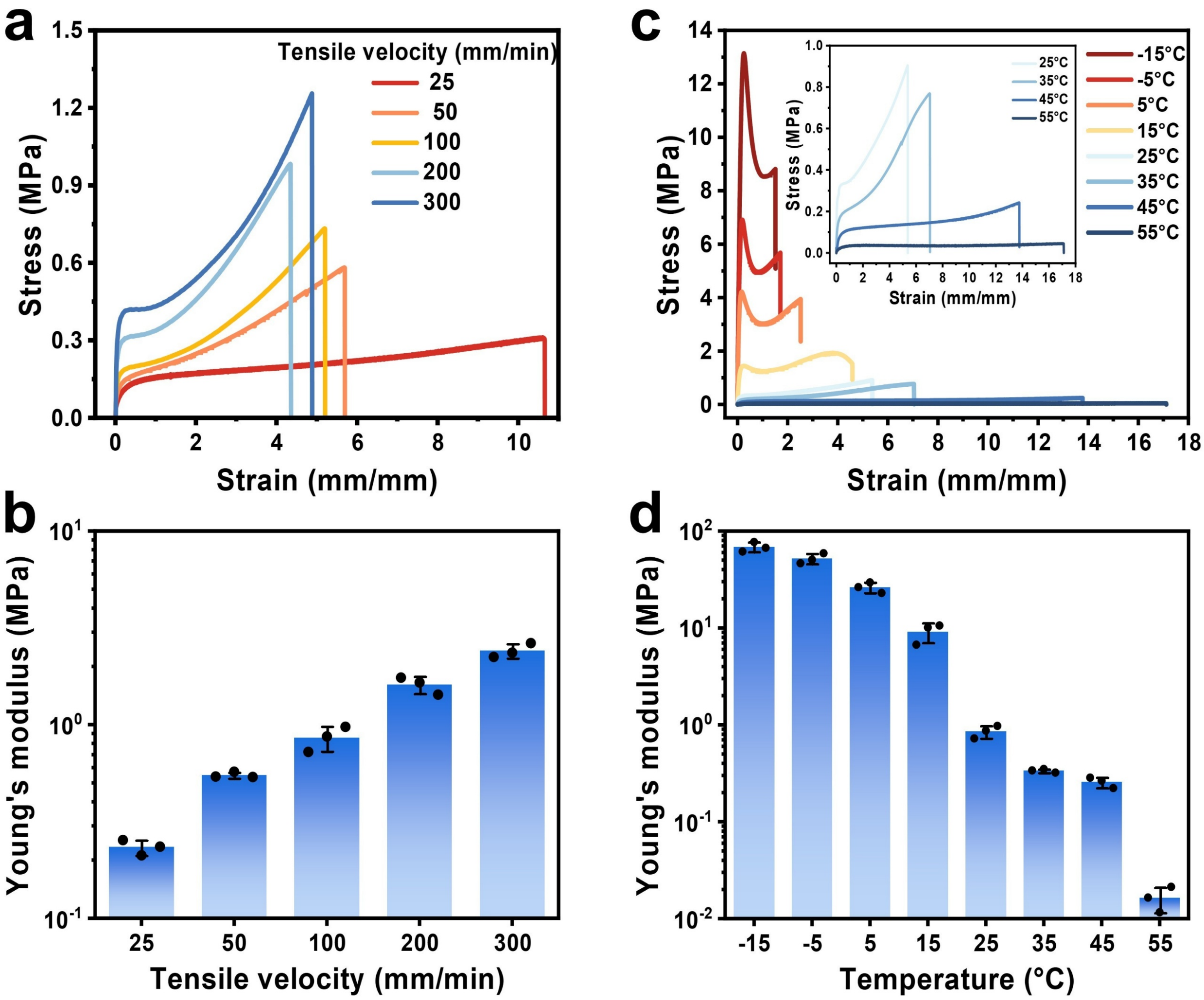

Supplementary Fig. 22. Rate- and temperature-dependent mechanical performance of the PEM$_2$ gel. **a** Tensile stress-strain curves at different tensile velocities. **b** Young's modulus and toughness values at different tensile velocities. **c** Tensile stress-strain curves at different temperatures. **d** Young's modulus values at different temperatures. Data are presented as mean values ± SD, n = 3 independent samples. Source data are provided as a Source Data file.

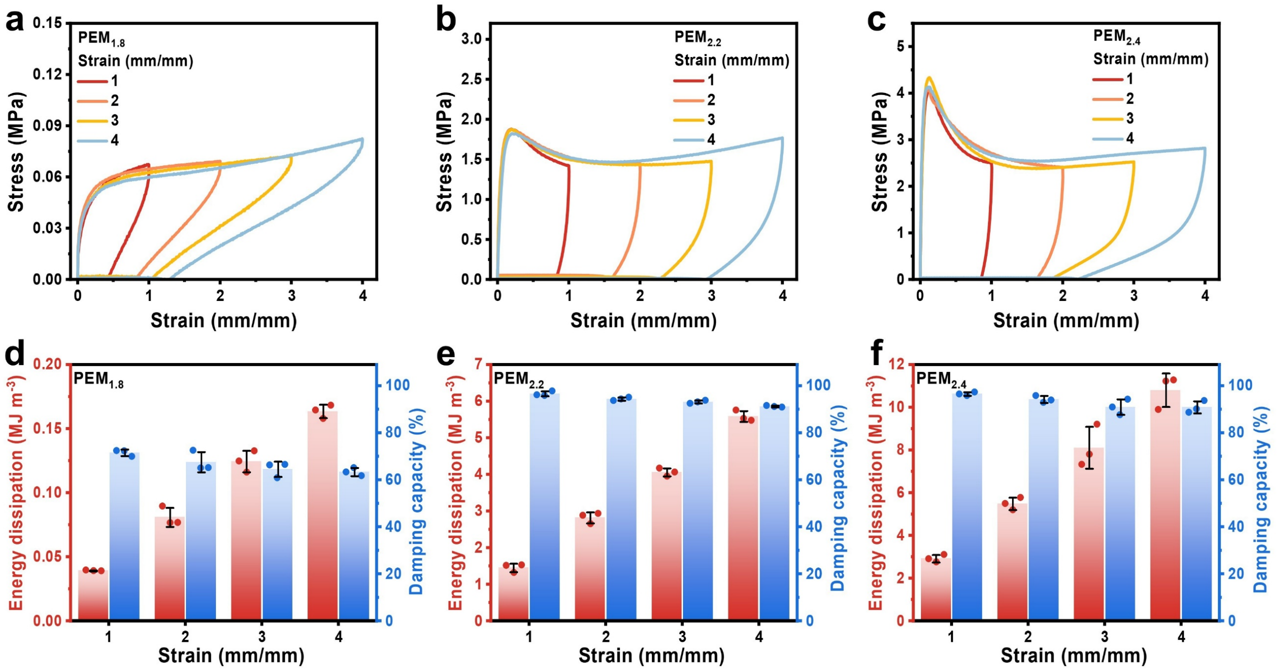

Supplementary Fig. 23. Energy dissipation and damping capacity of PEM$_y$ gel samples. a-c Tensile load-unload cycle curves of PEM$_y$ gel samples at different fixed strains. **d-e** Summary of energy dissipation values and damping capacity of PEM$_y$ gel samples with different tensile loading-unloading cycles. Data are presented as mean values ± SD, n = 3 independent samples. Source data are provided as a Source Data file.

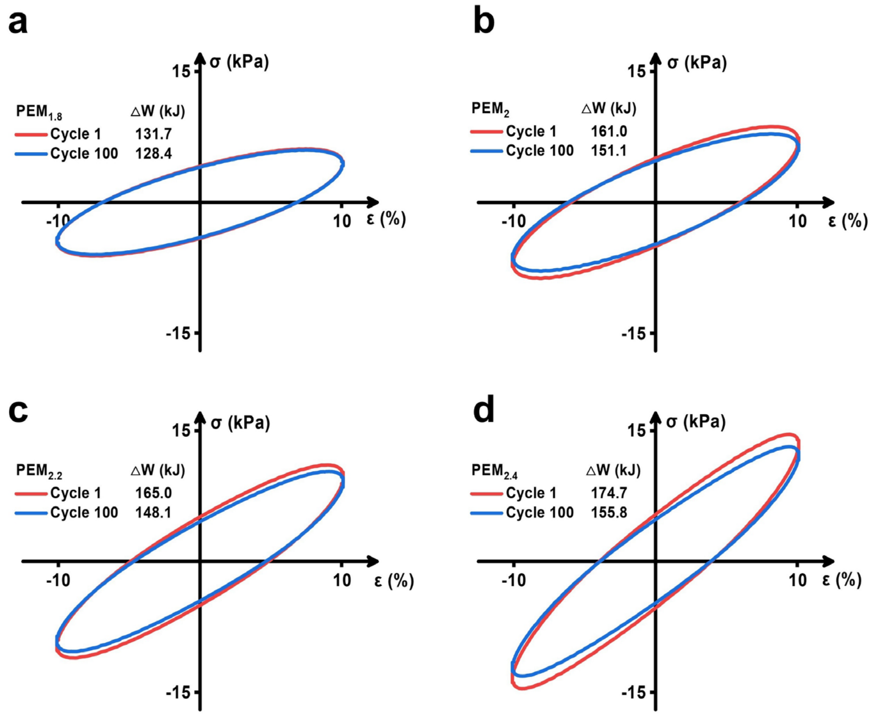

Supplementary Fig. 24. Large amplitude oscillatory shear (LAOS) tests of the PEM$_y$ gels. The hysteresis loops are elliptical, indicating all gels are located in the linear viscoelastic region at 10% strain[4,5]. The hysteresis loop area represents the mechanical energy converted into heat during each cycle and the area decreases only slightly after 100 cycles.

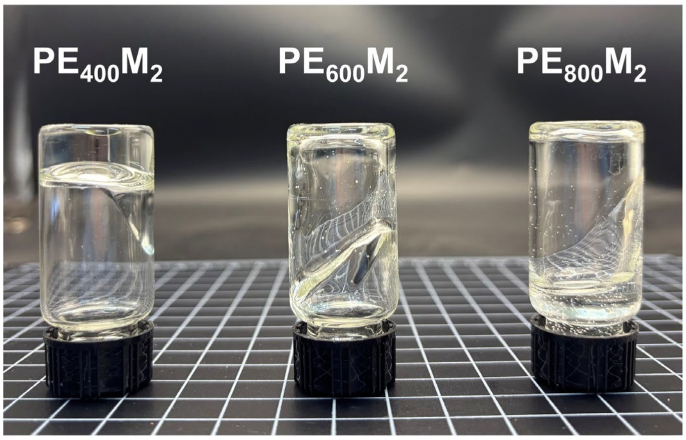

Supplementary Fig. 25. Photographs of $PE_{400}M_3$, $PE_{600}M_4$, and $PE_{800}M_5$ gels. The $PE_{400}M_2$ sample retains partial mechanical integrity and does not fully flow upon inversion, whereas the $PE_{800}M_2$ sample behaves entirely as a viscous solution. This progressive loss of structural integrity confirms that increasing the PEG chain length weakens density of strong hydrogen bonds and shifts toward weaker PMAA–PEG hydrogen-bonding.

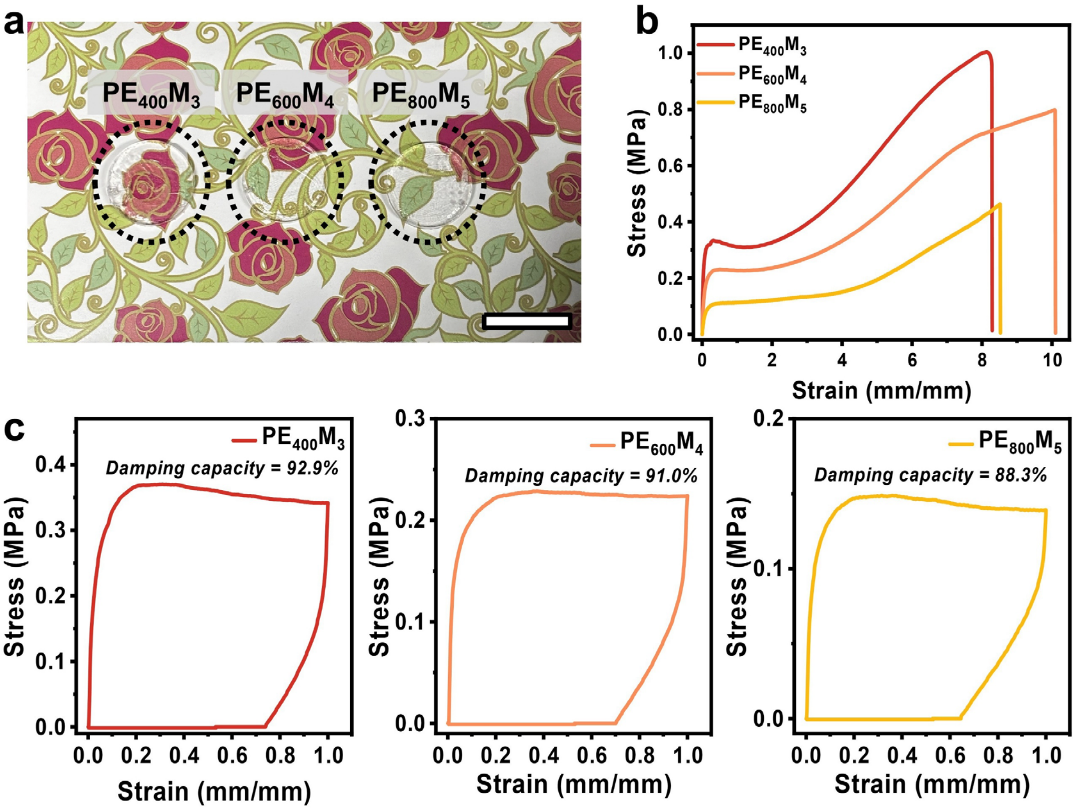

Supplementary Fig. 26. Verification of the universality of the oligomeric-solvent engineering strategy. a Photographs of $PE_{400}M_3$, $PE_{600}M_4$, and $PE_{800}M_5$ gels. Scale bar: 2 cm. **b** Tensile stress-strain curves of $PE_{400}M_3$, $PE_{600}M_4$, and $PE_{800}M_5$ gels. **c** Tensile loading-unloading curves of $PE_{400}M_3$, $PE_{600}M_4$, and $PE_{800}M_5$ gels at a fixed strain of 1 mm/mm, with damping capacity of 92.9%, 91.0%, and 88.9%, respectively.

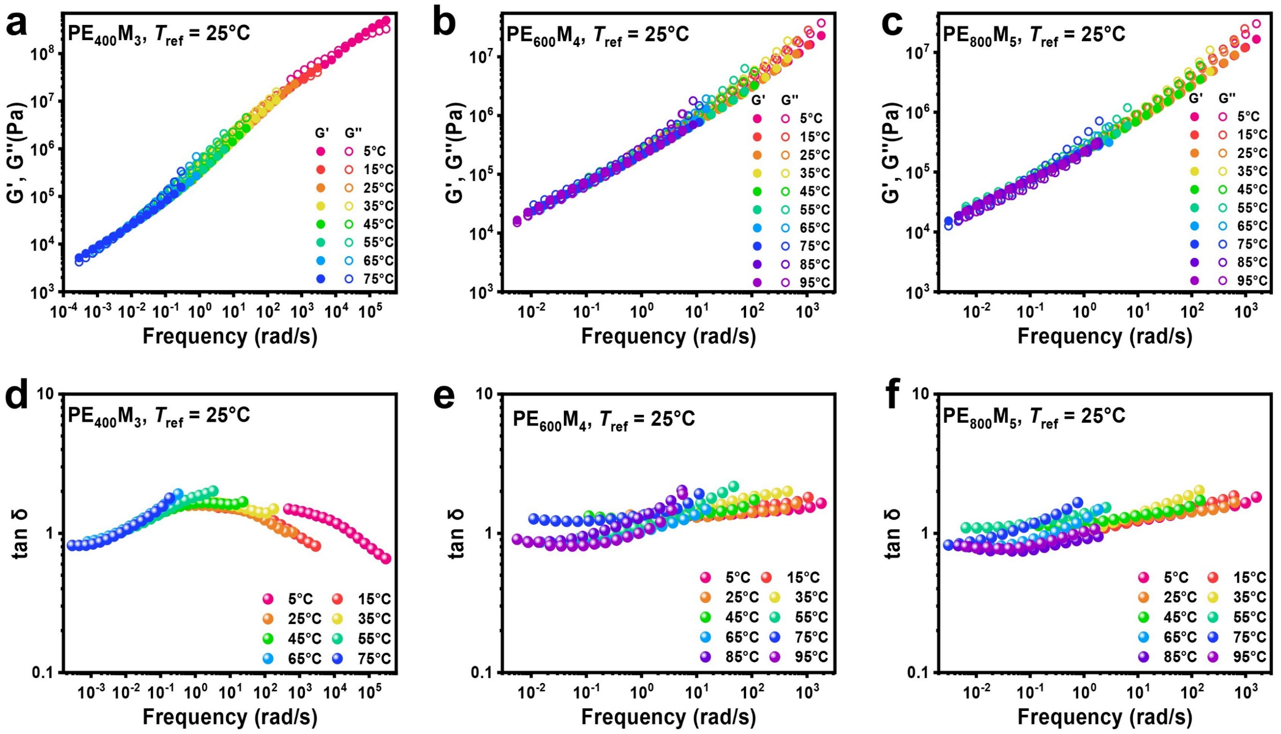

Supplementary Fig. 27. Rheological behavior of PMAA gels incorporating PEG of different molecular weights. a-c Rheological master curves of PE_xM_y gels constructed using the time-temperature superposition principle at a reference temperature of 25°C. **d-f** Loss factors of PE_xM_y gels obtained using the time-temperature superposition principle at a reference temperature of 25°C.

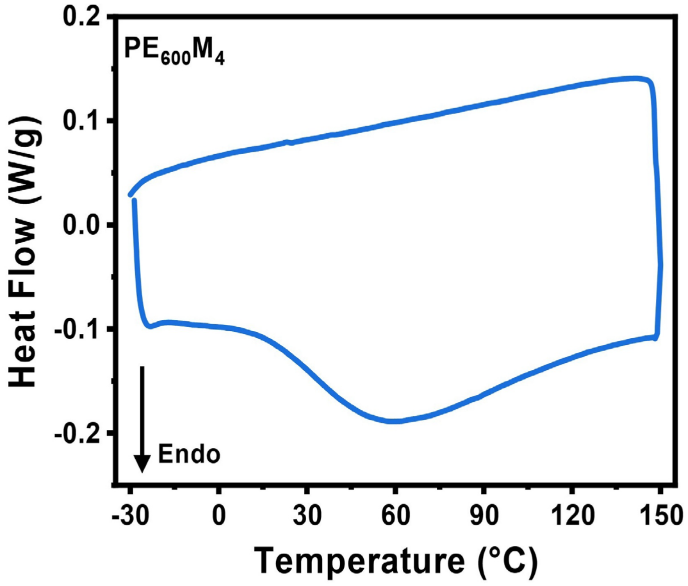

Supplementary Fig. 28. Cyclic DSC curves of the $PE_{600}M_4$ gel between -30 and 150 °C. The heating scan shows a clear endothermic peak, whereas no corresponding exothermic peak appears upon cooling.

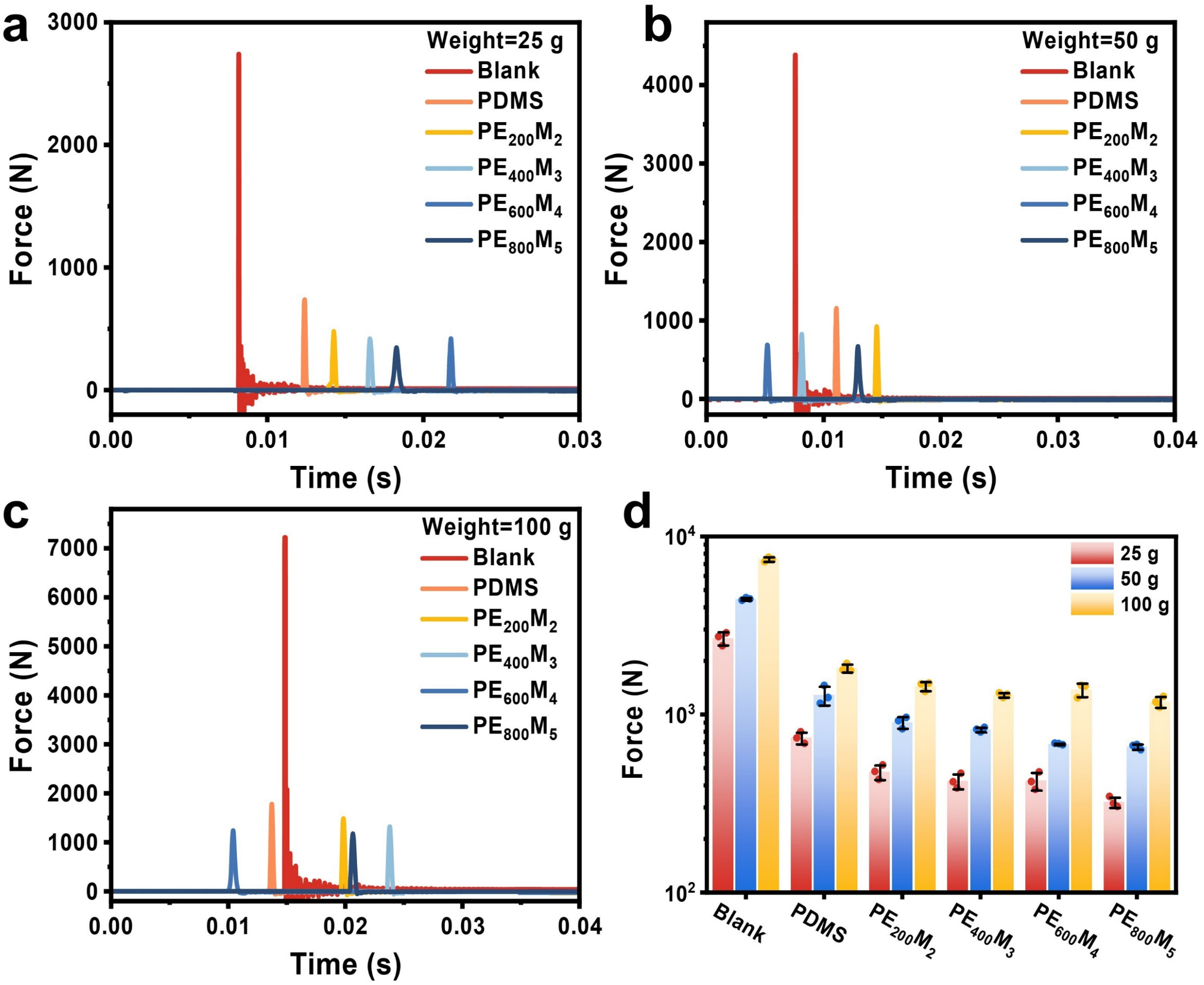

Supplementary Fig. 29. Force profiles during falling-ball impacts and the reduction of impact forces by PE_xM_y gel coatings with steel balls of different weights. Data are presented as mean values ± SD, n = 3 independent samples. Source data are provided as a Source Data file.

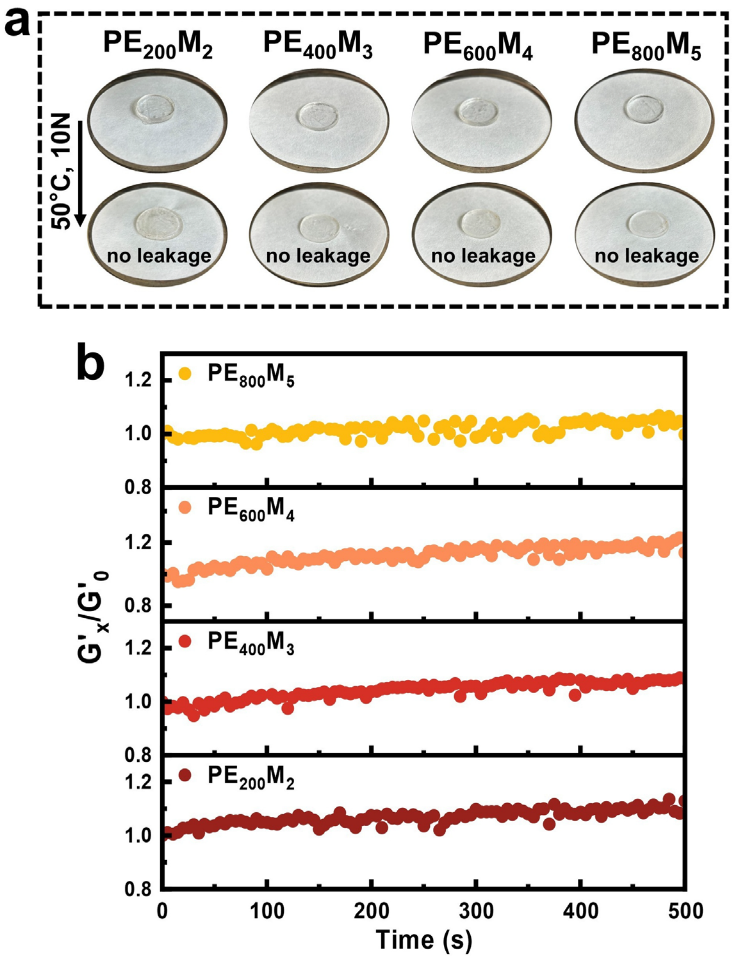

Supplementary Fig. 30. Assessment of solvent leakage in PE_xM_y gels. Temperature: 50℃, Pressure: 10 N, data were recorded every 5 s. a Stability test under pressure. **b** Dynamic mechanical properties of PE_xM_y gels during compression.

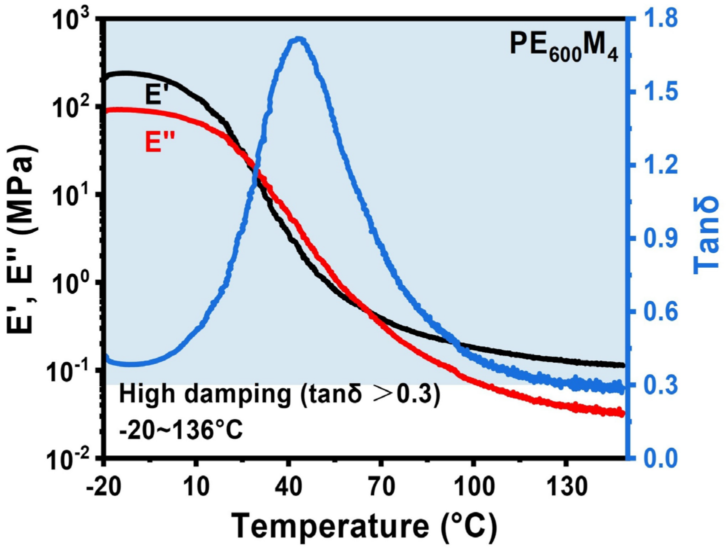

Supplementary Fig. 31. DMA results of $PE_{600}M_4$ gel between -20° and 150°C.

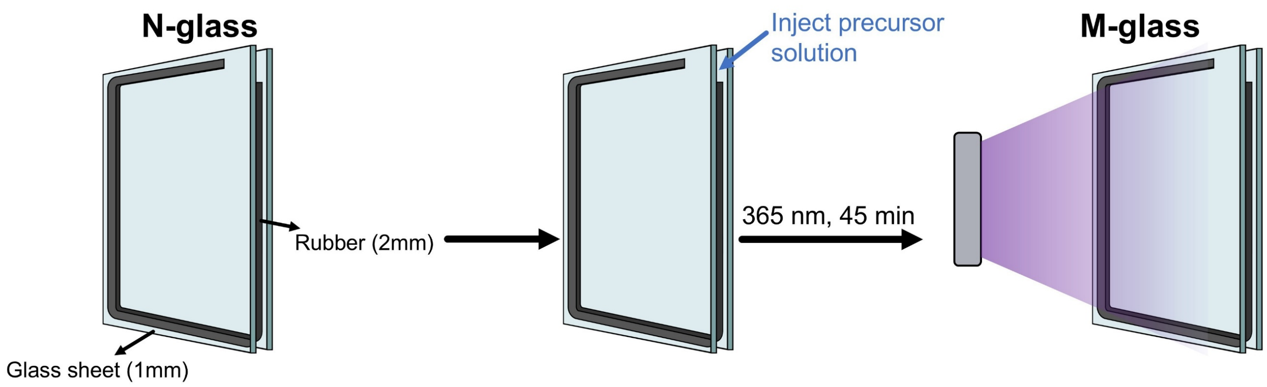

Supplementary Fig. 32. Illustration of the fabrication process of M-glass and N-glass. M-glass was prepared by sandwiching a 2-mm-thick silicone rubber spacer between two 1-mm-thick glass sheets, injecting the precursor solution, and curing it under UV light for 45 min. For comparison, conventional glass (N-glass) was fabricated in the same way, except that the interlayer was air.

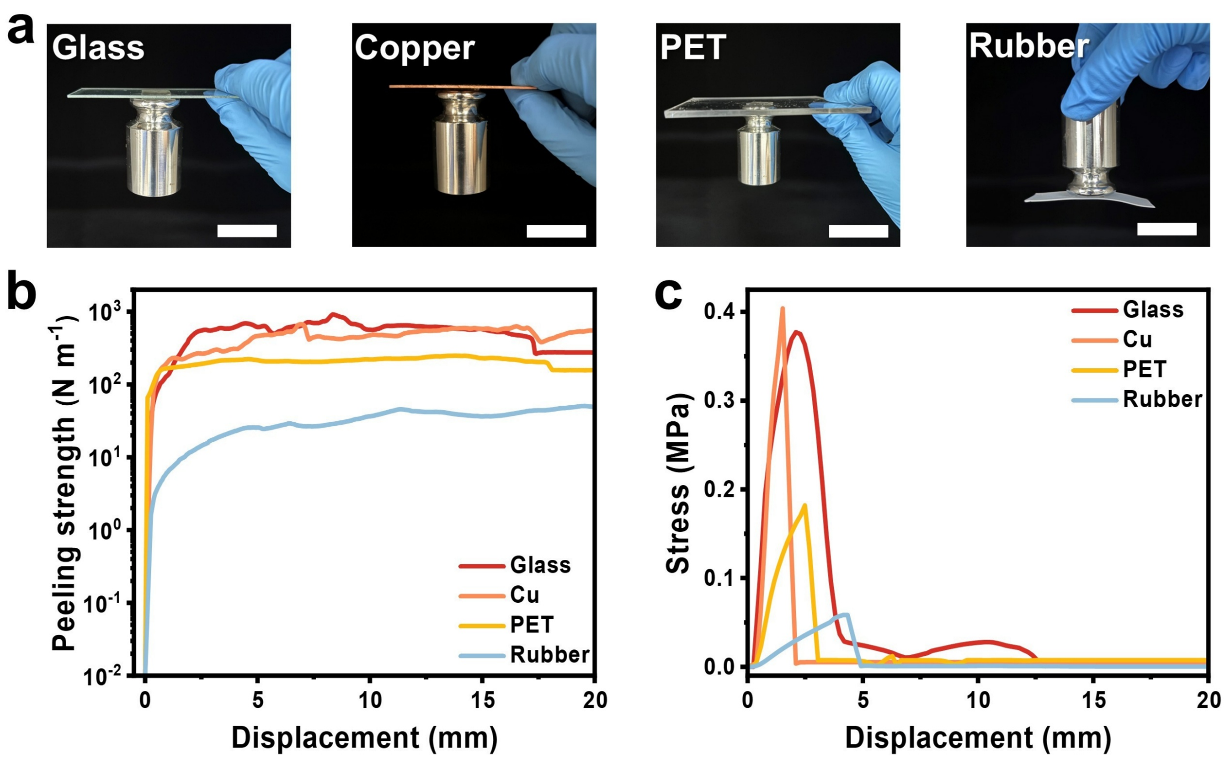

Supplementary Fig. 33. Adhesion performance of the $PE_{600}M_4$ gel. a Photographs demonstrating a square $PE_{600}M_4$ gel (1 × 1 cm^2) adhered firmly to various substrates. On glass, polyethylene terephthalate (PET), and copper, the adhesion was sufficient to readily lift a 200 g weight. Although adhesion on rubber was slightly weaker, it remained effective. Scale bar: 4 cm. **b** 90° peel curves of the $PE_{600}M_4$ gel on various substrates. **c** Shear adhesion stress-displacement curves of the $PE_{600}M_4$ gel on various substrates.

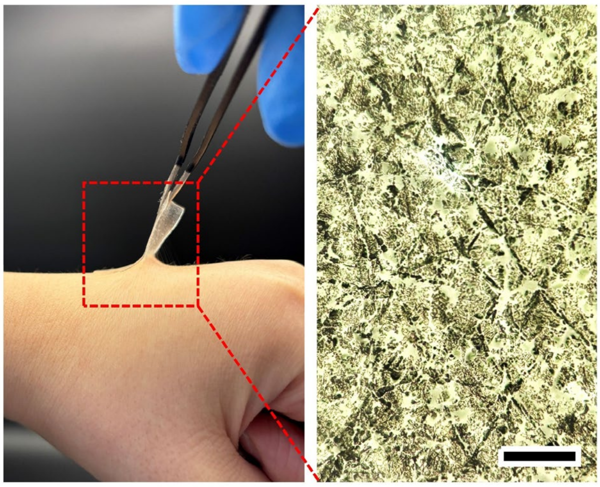

Supplementary Fig. 34. Photograph showing strong adhesion of the $PE_{600}M_4$ gel to human skin. Upon peeling the gel from the back of the hand, the surrounding skin was lifted concurrently, and the detached gel retained a clear imprint of the skin texture. Scale bar: 300 μm.

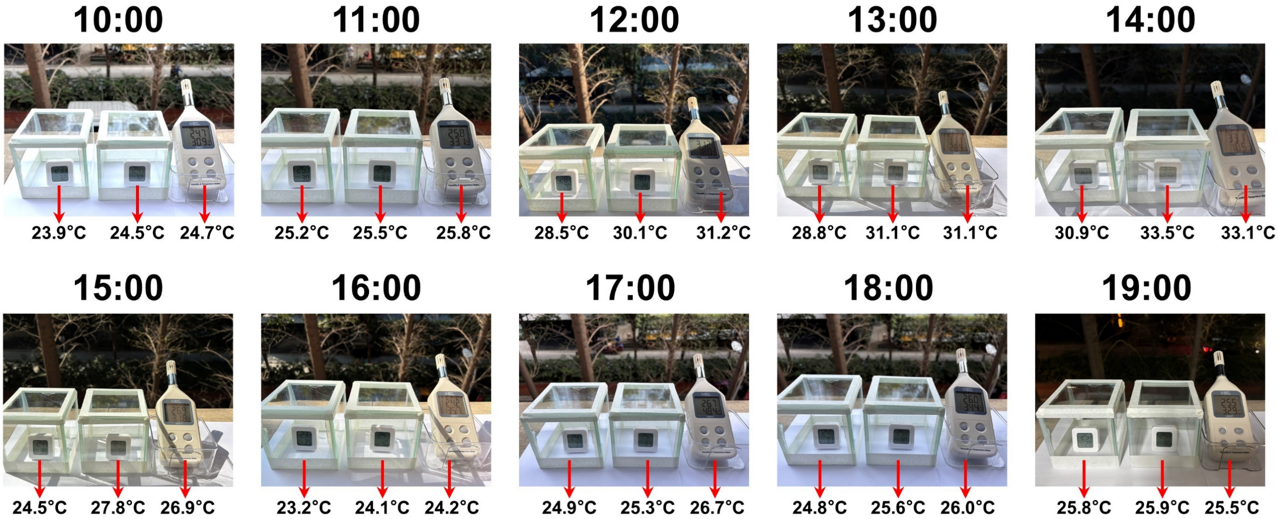

Supplementary Fig. 35. Photographs showing real-time temperatures of the blank group, the N-glass room, and the M-glass room from 10:00 to 19:00. From left to right: M-glass room, N-glass room, and blank group.

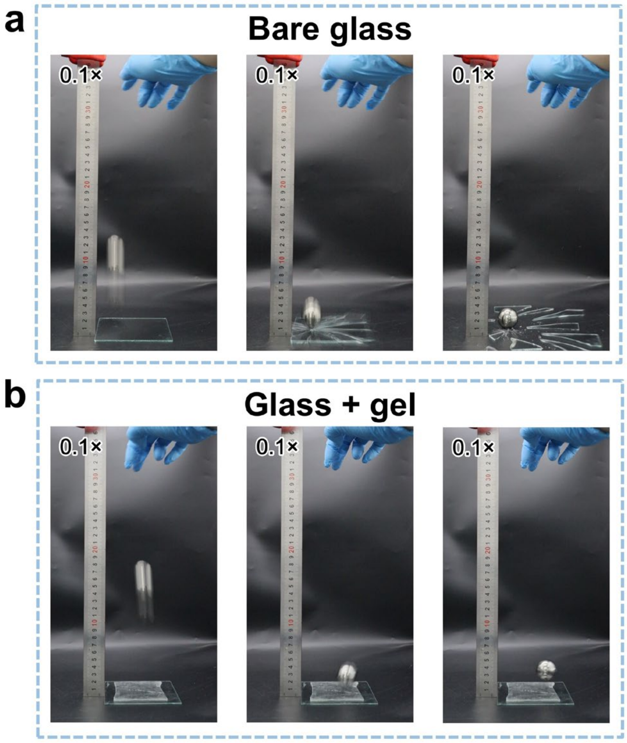

Supplementary Fig. 36. Comparison of impact resistance between glass with and without the PE$_{600}$M$_4$ gel protection layer. Upon impact by a falling ball, the bare glass fractured immediately, whereas the glass protected by the PE$_{600}$M$_4$ gel remained fully intact, with no detectable damage to either component. Any fracture observed in M-glass originates from stress concentration at the outer layer upon impact. In contrast, the excellent damping capability of the gel effectively dissipates the impact energy, preventing its transmission to the inner glass layer and thereby maintaining the structural integrity of the entire M-glass.

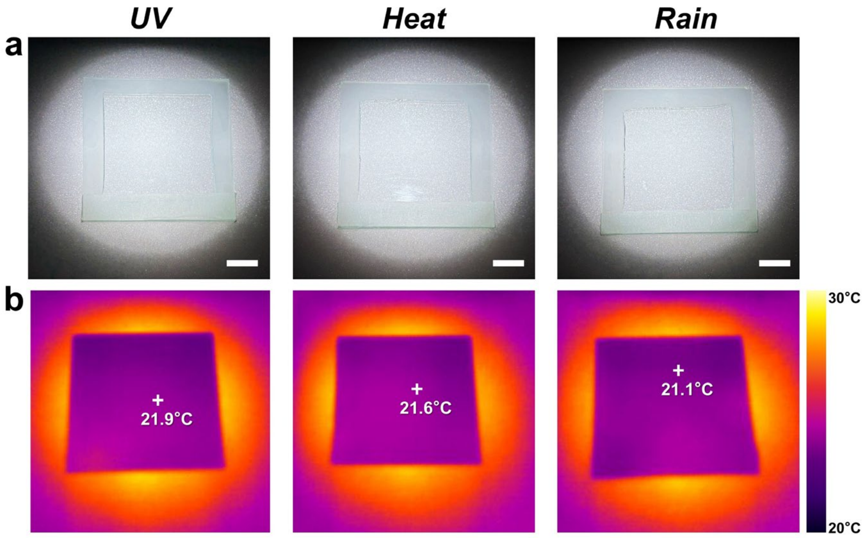

Supplementary Fig. 37. Thermal infrared images and photographs of M-glass under various environmental conditions. M-glass was irradiated under a UV lamp for 1 h, subjected to three heating-cooling cycles (heated at 40 °C for 2 h and cooled to room temperature), and rinsed under running water to mimic UV exposure, high-temperature conditions, and rain conditions, respectively. Scale bar: 2 cm.

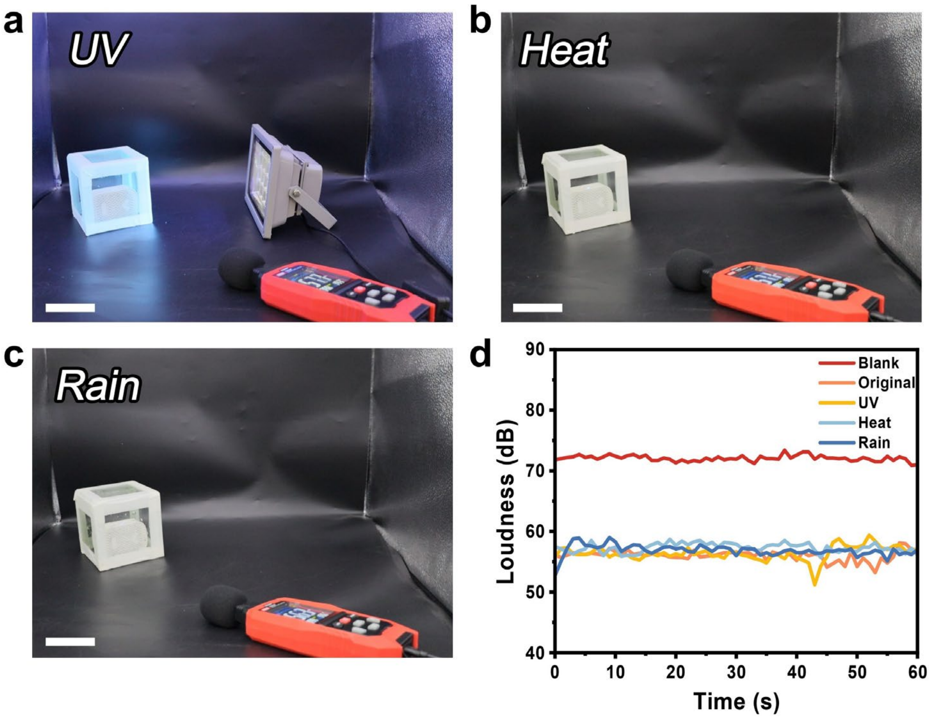

Supplementary Fig. 38. Noise-blocking capability of the M-glass in different environments. a-c Experimental setup for sound measurement under UV, heat, and rain conditions, respectively. Scale bar: 4 cm. **d** Loudness variation of the blank group, pristine M-glass room, M-glass room under UV , heat, and rain conditions.

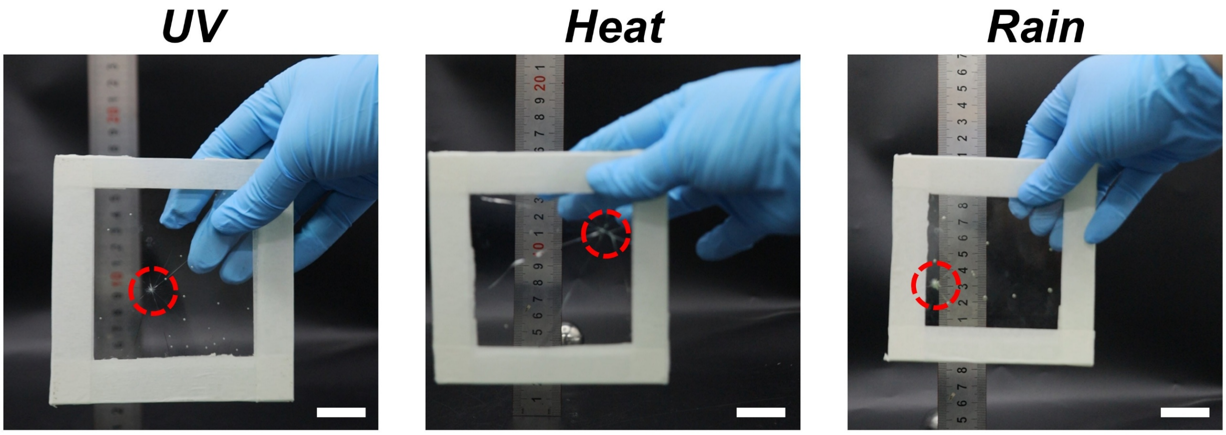

Supplementary Fig. 39. Photographs showing the M-glass under UV, heat, and rain conditions after impact by a 100 g steel ball dropped from a height of 30 cm. Scale bar: 2 cm.

Supplementary Table 1. Detailed compositions and formulations of the samples investigated in this work.

Sample code	PEG-200 (mol)	MAA (mol)	MBAA (mol/mol%) [a]	HMPP (mol/mol%) [a]
$PE_{200}M_{1.8}$	0.01	0.018	0.1%	0.2%
$PE_{200}M_{2}$	0.01	0.020	0.1%	0.2%
$PE_{200}M_{2.2}$	0.01	0.022	0.1%	0.2%
$PE_{200}M_{2.4}$	0.01	0.024	0.1%	0.2%
$PE_{400}M_{3}$	PEG-400 (mol)	MAA (mol)	MBAA (mol/mol%) [a]	HMPP (mol/mol%) [a]
	0.01	0.03	0.1%	0.2%
$PE_{600}M_{4}$	PEG-600 (mol)	MAA (mol)	MBAA (mol/mol%) [a]	HMPP (mol/mol%) [a]
	0.01	0.04	0.1%	0.2%
$PE_{800}M_{5}$	PEG-800 (mol)	MAA (mol)	MBAA (mol/mol%) [a]	HMPP (mol/mol%) [a]
	0.01	0.05	0.1%	0.2%
PEA	PEG-200 (mol)	AAc (mol)	MBAA (mol/mol%) [a]	HMPP (mol/mol%) [a]
	0.01	0.02	0.1%	0.2%
PMAA hydrogel	H_2O (mol)	MAA (mol)	MBAA (mol/mol%) [a]	HMPP (mol/mol%) [a]
	0.01	0.02	0.1%	0.2%

[a] Relative to MAA.

Supplementary Table 2. Comparison between the proposed in situ gel interlayer and conventional commercial glass interlayers.

Parameter	PVB	EVA	This work
Interlayer form	Polymer film	Polymer film	Liquid precursor
Construction method	Film stacking + autoclave	Film stacking + thermal curing	Direct injection + UV polymerization
Processing temperature	High (typically >120 °C)	Heating treatment (thermal crosslinking)	Room temperature
Processing pressure	High (autoclave required)	Low or none	None
Optical transparency	Achieved after thermal–pressure treatment	Achieved after thermal curing	Intrinsic from in situ polymerization
Interfacial adaptability	Limited (solid–solid contact)	Limited	Good (liquid wetting and in suit polymerization)
Manufacturing complexity	High	Moderate	Low
Equipment requirement	Autoclave	Heated laminator	Ultraviolet lamp

Supplementary References

1 Shi, Y., Wu, B., Sun, S. & Wu, P. Aqueous spinning of robust, self-healable, and crack-resistant hydrogel microfibers enabled by hydrogen bond nanoconfinement. *Nat. Commun.* **14**, 1370 (2023).

2 Zhang, X. N. *et al.* Influence of the α-Methyl Group on Elastic-To-Glassy Transition of Supramolecular Hydrogels with Hydrogen-Bond Associations. *Macromolecules* **55**, 7512-7525 (2022).

3 Zheng, Y. *et al.* Nanophase Separation in Immiscible Double Network Elastomers Induces Synergetic Strengthening, Toughening, and Fatigue Resistance. *Chem. Mater.* **33**, 3321-3334 (2021).

4 Ewoldt, R. H., Winter, P., Maxey, J. & McKinley, G. H. Large amplitude oscillatory shear of pseudoplastic and elastoviscoplastic materials. *Rheol. Acta* **49**, 191-212 (2010).

5 Huang, J. *et al.* Ultrahigh energy-dissipation elastomers by precisely tailoring the relaxation of confined polymer fluids. *Nat. Commun.* **12**, 3610 (2021).
