# Supplementary material for: Oligomeric-solvent engineering of hierarchical hydrogen-bonding networks for multifunctional glass interlayers
Source: Nat Commun. 2026 Mar 7;17:3607. doi: 10.1038/s41467-026-70223-7 (PMC13096342; doi:10.1038/s41467-026-70223-7)
Supplement: Supplementary file 2 — Description of Additional Supplementary Files [file 41467_2026_70223_MOESM2_ESM.pdf]

Description of Additional Supplementary Files

Supplementary Movie 1. Free-fall impact of a 25 g steel ball on glass with and without 3-mm-thick protective coating layers.

Supplementary Movie 2. Rebound behaviour of PDMS and PE600M4 gel balls dropped from a height of 30 cm.

Supplementary Movie 3. Demonstration of the strong interfacial adhesion of the PE600M4 gel formed in situ on glass.

Supplementary Movie 4. Sound reduction performance of different types of glass.

Supplementary Movie 5. Demonstration of the impact resistance of different types of glass.

Supplementary Movie 6. Demonstration of the sound-reduction performance of the M-glass under UV exposure, heating, and rainfall conditions.

Supplementary Movie 7. Demonstration of the distinct failure modes of bare glass and gel-protected glass under ball-drop impact.

Supplementary Movie 8. Demonstration of the impact resistance of the M-glass under UV exposure, heating, and rainfall conditions.
